# Changing knowledge and attitudes about childhood fever: testing a video instruction before its application in a health app

**DOI:** 10.3205/zma001546

**Published:** 2022-04-14

**Authors:** Moritz Gwiasda, Silke Schwarz, Arndt Büssing, Ekkehart Jenetzky, Hanno Krafft, Sara Hamideh Kerdar, Larisa Rathjens, Katja Boehm, David Martin

**Affiliations:** 1 Witten/Herdecke University, Chair of Medical Theory, Integrative and Anthroposophic Medicine, Herdecke, Germany; 2 Johannes Gutenberg University, Mainz University Medical Center, Department of Child and Adolescent Psychiatry and Psychotherapy, Mainz, Germany; 3 University of Tübingen, Department of Pediatrics, Tübingen, Germany

**Keywords:** fever, FeverApp, psychoeducational intervention, video clip, health app

## Abstract

**Background:**

Although infantile fever is harmless in most cases, many parents feel insecure when having to deal with it because important information is often missing. For educational purposes, an information video on fever in children was developed, which is also intended to serve as an onboarding element of a health app. The aim of the present work was to record the attitude of parents and adults on the topic of fever before and after the presentation of the information video, as well as its evaluation.

**Methods:**

Between May and November 2020, a total of 123 adults from three groups with different backgrounds (students, parents and educators) were interviewed using a questionnaire that was completed before and after the one-time presentation of an educational information video clip.

**Results:**

Several significant outcomes were recorded in attitude change toward managing fever, with no significant difference between groups. After viewing the informational video clip, 74% of participants would take body temperature rectally more often. In the after-questionnaire, 83% of participants agreed that they would now be more cautious about using fever-reducing medications. Before the video clip, 75% of the participants thought fever was “rather useful”; after, 93%. The level of temperature played a minor role as a reason for fever reduction. The information content and quality of the video were rated positively.

**Discussion:**

This study shows that a short information video is capable of bringing about a subjectively perceived intentional change in the attitude to dealing with fever, motivating a change in behavior, and reducing uncertainty in dealing with fever. Since the change in attitude was measured immediately after viewing the video clip, no statement can be made about the medium to long-term effect.

**Conclusion:**

The information video clip can be classified as a short-term fever education tool for which at least short-term effective is demonstrated. Long-term and possible synergistic effects when integrated into a health app with further information still needs to be investigated.

## Zusammenfassung

**Hintergrund:**

Obwohl kindliches Fieber in den meisten Fällen harmlos ist, fühlen sich viele Eltern im Umgang damit unsicher, weil oft wichtige Informationen fehlen. Zu Aufklärungszwecken wurde ein Informationsvideo über Fieber bei Kindern entwickelt, das auch als Onboarding-Element einer Gesundheits-App dienen soll. Ziel der vorliegenden Arbeit war es, die Einstellung von Eltern und Erwachsenen zum Thema Fieber vor und nach der Präsentation des Informationsvideos sowie dessen Evaluation zu erfassen.

**Methoden:**

Zwischen Mai und November 2020 wurden insgesamt 123 Erwachsene in drei Gruppen mit unterschiedlichem Hintergrund (Studenten, Eltern und Pädagogen) mittels eines Fragebogens befragt, der vor und nach der einmaligen Präsentation eines Aufklärungsvideos ausgefüllt wurde.

**Ergebnisse:**

Es wurden mehrere signifikante Ergebnisse in Bezug auf die Veränderung der Einstellung zum Umgang mit Fieber festgestellt, wobei es keine signifikanten Unterschiede zwischen den Gruppen gab. Nach der Vorführung des Informationsvideoclips würden 74 % der Teilnehmer die Körpertemperatur häufiger rektal messen. In der Nachbefragung stimmten 83% der Teilnehmer zu, dass sie nun vorsichtiger mit fiebersenkenden Medikamenten umgehen würden. Vor dem Videoclip hielten 75% der Teilnehmer Fieber für „eher nützlich“, danach waren es 93%. Die Höhe der Temperatur spielte als Grund für die Fiebersenkung eine untergeordnete Rolle. Der Informationsgehalt und die Qualität des Videos wurden positiv bewertet.

**Diskussion:**

Diese Studie zeigt, dass ein kurzes Informationsvideo in der Lage ist, eine subjektiv wahrgenommene intentionale Veränderung der Einstellung zum Umgang mit Fieber herbeizuführen, zu einer Verhaltensänderung zu motivieren und die Unsicherheit im Umgang mit Fieber zu verringern. Da die Einstellungsänderung unmittelbar nach dem Betrachten des Videoclips gemessen wurde, kann keine Aussage über die mittel- bis langfristige Wirkung getroffen werden.

**Schlussfolgerung:**

Der Informationsvideoclip kann als kurzfristiges Fieberaufklärungsinstrument eingestuft werden, für das zumindest eine kurzfristige Wirksamkeit nachgewiesen ist. Langfristige und mögliche synergetische Effekte bei Integration in eine Gesundheits-App mit weiteren Informationen müssen noch untersucht werden.

## Background

Regarding the use of digital health services, Germany is far behind in European comparison [https://ec.europa.eu/commission/presscorner/detail/de/MEMO_18_3737]. With the entry into force of the “Digital Health Care Act” (DVG) on December 19, 2019, the “app on prescription” for patients was introduced into health care. This means that approximately 73 million insured persons in the statutory health insurance system are entitled to a supply of Digital Health Applications (DiGA). There is a complex field of tension arising from the diverse possibilities of the technology and the different interests of the many stakeholders. At the same time, however, it is also becoming clear how great the benefits of mobile technologies are when they are used appropriately [1], especially in education regarding relevant topics and the possibility of increasing one's own health literacy. This is one of the goals of the “FeverApp” described below and is to be achieved, among other things, using multimedia content. In this app, an educational video clip is used whose “effectiveness” is to be tested in terms of information gain and intentional behavior change. Digital health education has the potential to achieve equivalent, or even better, learning effects than traditional learning methods [2].

Fever of one’s own child is one of the most common reasons for parents to consult the doctor [3]. Although infantile fever is harmless in most cases, many parents feel insecure and anxious when dealing with it [4]. At the same time, many parents often lack important information to take the best possible care of their child. Due to this uncertainty, more medication is often administered than necessary and children are presented to the emergency room more often than is actually necessary. With the help of the FeverApp, parents should receive information about the topic of childhood fever that is in line with the current state of science. The aim of the present study was to record general questions and attitudes about fever among parents/adults in comparison with students before and after the presentation of the information video clip, as well as its evaluation. Here, the primary endpoint was defined as the change in attitude towards fever. 

## Methods

### Study design

In the introduction of the FeverApp, parents are shown an informational video clip on the topic of fever in children as fever education. A before-and-after questionnaire was developed, which was completed anonymously by students, parents, and educators before and after the one-time showing of the informational video clip. Inclusion criteria included adults at least 18 years of age. These included students at a university (group 1), parents of kindergarten children (group 2), and educators at a vocational college (group 3). The survey on the video clip was conducted accordingly in three groups, which were combined into an overall collective at the end. During the COVID-19 restrictions, the surveys had to take place online. The primary endpoint of the study was to compare attitudes toward fever before and after the video; this was operationalized by 22 knowledge questions from three categories (measurement location, warning signs, and fever reduction).

#### Description of the information video clip

**Figure 1:**
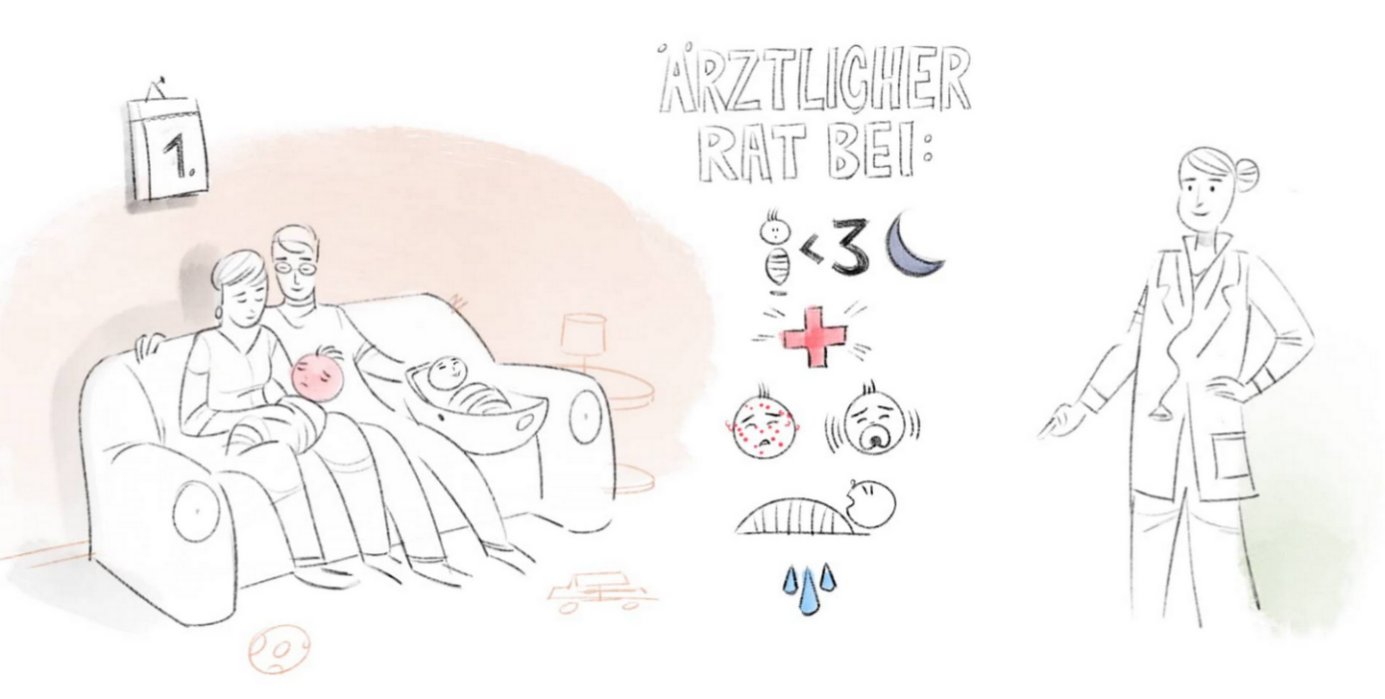
Still from video clip, recommendation when to seek medical advice

The 4:19 minute video clip [https://www.feverapp.de/video] shows information about fever in cartoon style (see figure 1 (Fig. 1)). At the beginning, it is pointed out that the information shown comes from pediatricians and adolescent doctors. It is explained that fever is a normal immune reaction, followed by a list of warning signs where medical advice should be sought. In addition, the possibility of febrile convulsions is discussed, as well as the fact that they do not last long, do not involve any sequelae, and cannot be prevented with fever reduction. This is followed by information about the need for attention and a chance to build a relationship with a sick child. Recommendations are given on naturopathic measures and that fever-reducing drugs and antibiotics are not always necessary and should only be used in consultation with the doctor. It is reiterated that there is no maximum temperature limit above which fever should be reduced and that temperatures above 40°C can be well tolerated. Finally, reference is made to medical advice and the FeverApp and its benefits are briefly presented. 

#### Intervention and questioning

The prior questionnaires (see attachment 1 ) asked general questions about the management of fever in children and about the evaluation of the short video clip. The latter collected demographic data (7 items) and general management of fever (15 items). These included questions such as “At what temperature do you speak of fever?” or about the preferred measurement location. This was followed by questions on the means used to deal with fever, as well as the attitude towards fever reduction, such as “What are your reasons for reducing fever?”. The informational video clip was then shown. This was followed by the post-survey (see attachment 1 ), which repeated 14 items on the general approach to fever from the before questionnaire, as well as another 20 items to evaluate the video clip and 3 items to inquire about further interest in an app on fever. The evaluation of the video clip included free text fields in which newly learned information, as well as open questions and additions were to be entered, as well as questions with various statements about the structure and content of the video clip, such as “The information on the subject of fever is easy to understand”. The total time required to answer the questions, including watching the video, was approximately 15-25 minutes.

#### Statistical methods

Most of the before-after surveys were conducted by means of multiple choice, and accordingly a nonparametric procedure for nominal data of a dependent sample was selected for this purpose in the form of the McNemar-Chi-Quardrat test; in addition, the alpha error cumulation was neutralized with the aid of the Bonferroni correction. The case number calculation for a two-sided test in the McNemar Chi quardrat test according to Conett et al., results: With a global error probability of 5% and a power of 80% and the assumption that 10% switch from affirmative to negative and 25% vice versa, one needs a total of 118 subjects participating in both surveys. Due to the 22 questions to be answered (multiple testing), the significance limit of the global alpha was set from 0.05 to 0.00227 for the single knowledge aspect. For temperature data, mean comparisons could be applied using a T-test for connected samples. In each case, the tests were performed at a significance level of p>0.05, and all other conditions were met. The evaluation of the video was evaluated purely descriptively, since it was not comparative data. The free texts were evaluated with the help of a summary mask, in which the frequencies of the mentions of similar statements were also considered. 

## Results

### Demographic data

The first group included n=45 students from Witten-Herdecke University, 30 of whom were female and 15 male; their average age was 24±5 years. Three of the participants (TN) had children. Most of the TN were studying human medicine (n=21), other frequently mentioned courses were management (n=9) and psychology (n=8). On average, the TN were in their 3^rd^ or 4^th^ semester of study. In the group of educators (n=62), there were 51 women and 11 men, and the average age was 28±9 years. In the group of parents (n=16), there were 14 women and only 2 men, and the mean age was 38±6 years. There were no significant differences between the groups for gender distribution, but they differed significantly for age. The latter groups can be combined into one group of “older” adults (n=78) with a higher proportion of individuals with children of their own. Of these, 65 were women and 13 were men; their average age was 30. A large proportion of the TN were with partners, either unmarried with a partner (n=24), or married (n=20). A total of 29 of the TN had children. The total number of all TN was N=123. Global testing between groups revealed no significant differences in response behavior, so the groups were combined into an overall collective.

#### Attitude to fever

For the question “At what temperature in °C would you speak of fever?” a significant (p<.001) difference is found in the response behavior before (38.2°C) and after (38.5°C) the video.

Responses to the question “Where would you take temperature?” changed significantly in the overall collective (N=123): after the video intervention, most (74.8%) TN would now be more likely to take temperature rectally, this number more than doubled compared to before (29.6%). All other measurement sites also decreased proportionally: the proportion who would measure orally decreased from 40.8% to 24.4%, as did axillary from 31.2% to 15.1% and auricular from 45.6% to 21.0%. However, after Bonferroni correction, only the changes in the auricular measurement location were significant.

After watching the video, the TN would be more likely to go to the doctor in case of corresponding symptoms in combination with fever (see table 1 (Tab. 1)), the most significant increase is for the symptom “skin rash”, overall the increase is slight, but significant in some cases.

This was followed by two questions on the topic of fever reduction. The participants were first asked whether they would use naturopathic or similar medications to reduce fever. Before the video, 35% would use naturopathic or similar medications to reduce fever; the number increased to 45% after the video. The question whether the participants treat malaise during fever with other means (calf compresses, etc.) was answered in the affirmative by 68% of the participants before the video, this number increased to 87%; subsequently, calf compresses were mentioned here in particular. In the post-questionnaire, a total of 83% of the participants agreed with the statement that they would now be more cautious with the unauthorized “use of fever-reducing drugs such as paracetamol or ibuprofen”. 

Most TN (n=84) had made (drug) fever reduction dependent on body temperature before the video, whereas after the video the number of these TN decreased (n=52). A total of 45 participants made fever reduction dependent on “other criteria” before the video, while this number rose to 58 after the video. Among these “other criteria”, “feeling well” and “accompanying symptoms” were mentioned most frequently in the free text field. Among the reasons for fever reduction, the most frequently mentioned were “avoidance of consequential damage due to excessively high temperatures”, “avoidance of febrile convulsions”, and “avoidance of brain damage due to febrile convulsions” (see table 1 (Tab. 1)).

Before watching the informational video clip, 93 TN thought fever was “rather useful”; after watching the video, 115 did. Beforehand, 106 TN answered the question “When would you reduce fever in your child?” with a temperature response that had a mean (MW) of 39.1°C (6 TN gave other reasons in free text, such as “When the child is very unwell”). After the video clip, the response of 78 TN to the question about body temperature of fever reduction differed significantly (p<.001) with a MW=40.0°C (here, 28 gave other reasons, the most frequent being “feeling well”, as well as “after consulting a doctor/physician”). The knowledge of what to consider and do when dealing with fever was shaped in 100 TN by their family.

#### Video evaluation

In the free text entries on newly learned information, the participants stated that they had learned from the video clip that

1. high body temperatures can also be well tolerated (n=22),
2. fever is an initially positive body reaction to infections (n=19) and,
3. that fever-reducing medication or fever reduction in general can also have negative effects, i.e. fever does not always have to be reduced immediately (n=15).

The open question whether there were still questions about fever after watching the video was answered by 31 TN. The most frequent question of the participants was from which temperature level fever is defined (n=6), as well as whether there is a fever temperature that is alarming/dangerous (n=4). The evaluation of the information content and the quality of the video was predominantly positive. Most clearly, the TN agreed with the statement that the information in the clip is “well understandable”, as well as “present to a good degree”, as well as clarified by the “use of graphics and text [...] meaningful” (see table 2 (Tab. 2)).

In a free text question, suggestions were made for improving the audio quality, changing the speed and adding a summary of the information at the end. In the free text question about what else the TNs would add to the video, there were very heterogeneous statements about the pace of the information. Some TNs wanted a faster pace and felt underwhelmed (however, this comment came largely from medical students), while another comment indicated just the opposite. 

The question “Did the clip change anything in your attitude towards fever?” was answered yes by 58% of the TN; in the corresponding free text field “Yes and indeed” mainly knowledge about symptoms and behavior in fever, as well as a more relaxed attitude towards fever and less fear were mentioned; the latter in the sense that “fever can be seen as something positive”. Overall, 80% of responding TNs indicated basic interest in a health app on the topic of fever. When asked what the participants would use such an app for, they indicated “safety in dealing with fever/symptoms”, “valid information about fever”, “warning of possible dangers and recommendations for action (e.g. visit to the doctor/physician)”.

## Discussion

The study shows that a short informational video clip is able to bring about an intentional change in attitude towards the management of fever. The effect of the video is reflected in the significant changes in the responses, as well as in the comments of the free text fields. However, an overall score in the sense of a primary endpoint cannot be formed for this, since there is no weighting basis of the individual items to be aggregated, accordingly they are considered individually and categorically. In 58% of the TN, the video intervention led to a general change in attitude towards fever, 83% would now be more reluctant to “use antipyretic drugs such as paracetamol or ibuprofen” on their own initiative, and fever was finally rated as rather useful by almost all (98%). The TNs also developed a greater awareness of the symptoms for which medical consultation is necessary. Overall, TNs were less likely to reduce fever quickly, and if they did, they were also significantly more open to using less invasive home remedies to reduce fever or increase comfort, such as calf wraps or foot baths. Antipyretics, which are often used too uncritically [5], would be used much more cautiously after the video clip. Fear of fever can lead to significantly increased and unnecessary use of antipyretics and antibiotics, thus having negative effects on the child [4]. The video clip shown could be helpful in resolving and possibly preventing the often-unfounded fear of fever. One advantage for physicians is the time saved in educating parents about childhood fever through the use of the video clip: they could also refer to the video in the educational discussion as an opportunity to refresh and deepen what was said in the consultation, at anytime and anywhere especially because the survey showed that most participants have their experiences with fever from their own family, so they were shaped there with their attitudes and behaviors, special attention should be paid here to fever education. 

In recognizing possible warning signs, significant changes can be seen in some cases: before the video, 72% of the TN would have sought medical advice for “skin rash”; this figure increased to 89%. However, other studies have shown that the educational use of media such as videos alone is not very effective; instead, they would need to be embedded in an educational program [6]. Video is more of a technology used to deliver content rather than a content body per se. Some researchers emphasize that videos must be used with a clear purpose in mind to support learning [7], .[8] Thus, a video will only reach its full potential in a well-designed learning environment [9]. Research suggests that the prior learning goal and purpose should determine what type of delivery strategy is used when embedding educational video clips [6]. While the goal of novel educational tools and interventions is to enhance the acquisition of knowledge and skills, it is equally important that these enhancements “stick” or have a longer-term impact [10]. Using established principles, such an educational intervention seeks to create a tool that is contextual, relevant, easily accessible, engaging, self-directed, and includes social interaction [11]. The educational video can thus be an effective onboarding element for a health app that aims to provide a more complex and in-depth exploration of the topic of fever.

It is interesting to note that there was no significant difference in the increase in knowledge between the groups. It is possible that the video clip can convey its information content regardless of the previous education of the audience, which would need to be verified with the participation of people from further educational levels. Since this study asked, among others, young students, almost half of whom (47%) are in advanced semesters in the field of human medicine, it could be assumed that this group has an above-average health literacy. Nevertheless, an increase in knowledge could also be recorded among the medical students, just as in the other groups. Only three out of 45 students had children, which could also have an effect on the response behavior. For the other two groups, it should also be noted that most of the TN were childless, with a total of only 29 parents among the TN. The recruitment of parents for this study proved to be particularly difficult, as contact restrictions due to the Corona pandemic meant that no parents' evenings took place at schools and kindergartens, where they were originally supposed to have taken place.

The video was positively evaluated by the participants. The pace of the video was designed to make the information accessible to as many people as possible. Since many of the participants presumably have a high level of health literacy through their studies, the increase in speed desired by some medical students could have a negative impact on the accessibility of the video for people with lower health literacy. In this case, the inclusion of additional groups would be useful.

The change in attitude was recorded immediately after viewing the video. Here it would be interesting to see whether this also remains constant over a longer period of time. However, it should be noted that the video clip should be viewed as one component of a more complex health app, which was studied in isolation in this case. It can be assumed that parents will take advantage of the opportunity to watch the video clip repeatedly as needed, thus reinforcing the learning effect. Especially if the information thematized in the video clip, in the case of a feverish child, can be applied immediately. The video clip was thus viewed in a neutral test situation, while it may be that parents with acutely feverish children would absorb the information offered with increased attention. The app already offers the video clip during onboarding. In case of fever, it can be watched again. In the future, further observation of the parents' behavior metadata within the app will be of interest in order to discuss in which specific situations the video is used and whether any further effects are discernible, as well as to verify the previously mentioned statements.

### Limitations 

Under the conditions of the COVID-19 pandemic, recruitment of subjects was extremely difficult, which meant that the number of TN in the subgroups was not large enough to illuminate representative comparisons between the groups in depth. Accordingly, these had to be combined into an overall cohort, reducing the breadth of possible conclusions. 

The effect of the video was measured immediately after the first viewing, this is a narrow sample and does not allow statements about a possible long-term effect, which may well be relevant when asking about attitude change. In addition, the attitude is asked purely by self-assessment, which entails a structural subjectivity, a possibility to balance this would be behavioral observation in a controlled setting, which was not possible under the given circumstances.

## Conclusions

In summary, the video clip on the management of fever provided a significant gain in knowledge and an intentional change in attitude. Thus, a tool was created that successfully communicated contextually relevant information related to childhood fever. The educational video clip is easily accessible, engaging, and subject to self-direction, allowing it to be accessed whenever needed. It can be described as an effective fever education tool. The synergistic effect by integrating it into a health app with further information should be further explored.

## Funding

The funding is composed of: 20% Software AG Foundation [https://www.sagst.de/]; 80% Federal Ministry of Education and Research (BMBF) (Fkz: 01GY1905) [https://www.gesundheitsforschung-bmbf.de/de/fieberapp-register-aufbau-eines-registers-zur-information-und-selbstdokumentation-der-9014.php]. 

All described studies and evaluations were performed with the approval of the responsible ethics committee of the University of Witten/Herdecke (application no. 139/2018), in accordance with national law, and in accordance with the Declaration of Helsinki of 1964 (in the current, revised version 2013).

## Competing interests

The authors declare that they have no competing interests. 

## Supplementary Material

Questionnaire Health Clip (shortened version)

## Figures and Tables

**Table 1:**
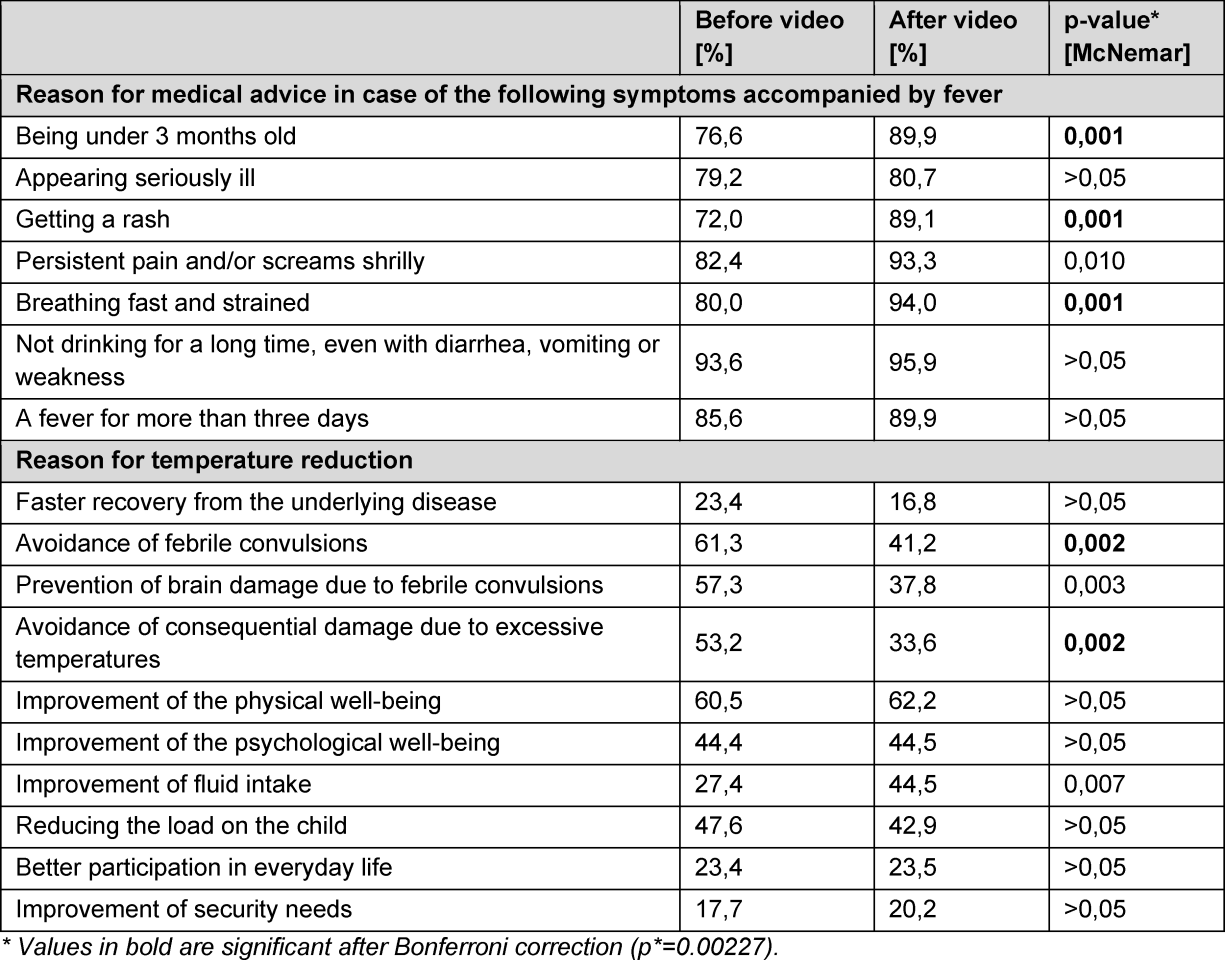
Percentage of responses to the questions “If the following symptoms occur together with fever, seek medical advice” and “What are your reasons for reducing fever?” from the before and after questionnaires, multiple responses (N=123)

**Table 2:**
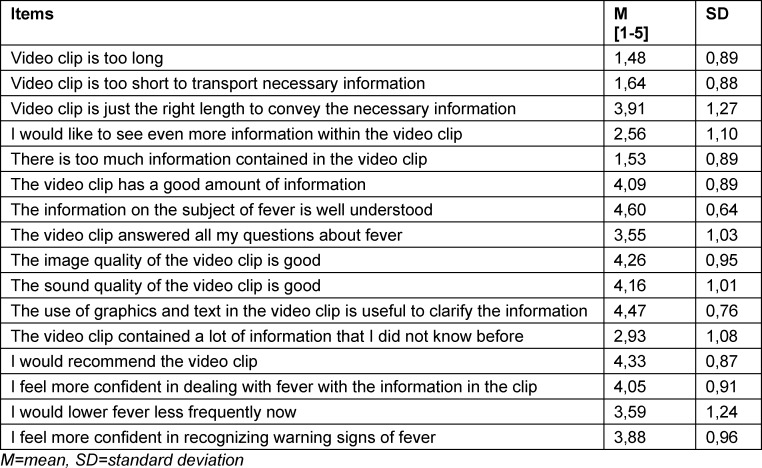
Means (MW) and standard deviation (SD) of responses to statements about the video in a matrix from the questionnaire about the video clip, 5-point Likert scale (1-dont agree at all to 5-agree completely) (N=123).
