# Supplementary material for: Changing knowledge and attitudes about childhood fever: testing a video instruction before its application in a health app
Source: GMS J Med Educ. 2022 Apr 14;39(2):Doc25. doi: 10.3205/zma001546 (PMC9174070; doi:10.3205/zma001546)
Supplement: Questionnaire Health Clip (shortened version) [file JME-39-25-s-001.pdf]

Attachment 1: Questionnaire Health Clip (shortened version)

Please note that a shortened version of the original questionnaire (in German) is provided. Since the video is only available in German, the questions related to the video have not been included in the English version.

Dear Parents,

As part of a research project at the University of Witten/Herdecke, we would like to ask you to participate in an anonymous survey (i.e. without name, address or other identifying details).

This involves general questions about fever, as well as the evaluation of a short video clip. This will help us to develop an app that can accompany and support parents with children in dealing with fever can accompany and support.

Kind regards

Prof. Dr. med. David Martin

Dr. med. Silke Schwarz

Your participation is voluntary. You are free to discontinue your participation at any time, without any disadvantages for you. Your data will be stored anonymously. The means that the information you provide in the study cannot be linked to you personally. The data are only accessible to the researchers involved in the study and are never passed on to third parties and will not be passed on to third parties at any time. The data that you enter anonymously will be handled in accordance with DSGVO compliant.

There are 42 questions in this survey.

Demographics

Information about yourself

Your gender

Please select only one of the following answers:

- Female
- Male
- Diverse

Your family status
Please select one of the following answers:

- married with partner
- unmarried with partner
- without partner

Your age in years
Only numbers may be entered in this field.
Please enter your answer here:

-

Number of my children
Please select one of the following answers:
Please select only one of the following answers:

- 0
- 1
- 2
- 3
- 4
- 5
- 6
- Other

Please state the age of your children in years
Answer this question only if the following conditions are met:
Answer was NOT "0" to question "4 [NumberChildren]" (Number of my children).
Only numbers may be entered in these fields.
Please enter your answer(s) here:

- 1st child
- 2nd child
- 3rd child
- 4th child
- 5th child
- 6th child

Your highest general education degree
Please select one of the following answers:
Please select only one of the following answers:

- Hauptschule
- Secondary school
- High school diploma
- Technical college
- University

Your nationality
Please enter your answer here:

Attachment 1 to Gwiasda M, Schwarz S, Büssing A, Jenetzky E, Krafft H, Hamideh Kerdar S, Rathjens L, Boehm K, Martin D. *Chaning knowledge and attitudes about childhood fever: testing a video instruction bevor ist application in a health app.* GMS J Med Educ. 2022;39(2):Doc25. DOI: 10.3205/zma001546

Fever 1

From what temperature in C° would you speak of fever?
Only numbers may be entered in this field.
Please enter your answer here:

Would you take the temperature?
Please select the answers that apply:
Please select all that apply:

- no
- rectally (in the buttocks)
- oral (in the mouth)
- axillary (in the armpit)
- in the ear

If the following symptoms occur together with fever, medical advice should be sought:
Please select the answers that apply:
Please select all that apply:

- if the child is under 3 months old
- if the child seems seriously ill
- if the child develops a skin rash
- if the child has persistent pain and/or shrill cries
- if the child is breathing fast and labored
- if the child does not drink for a long time, even with diarrhea, vomiting or weakness
- if the child has a fever for more than three days
- Other:

What do you do if your child has a fever?

Does your child attend daycare, kindergarten, or school when he or she has a fever (after fever reduction, if necessary)?
Answer this question only if the following conditions are met:
Answer was NOT '0' to question '4 [numberchildren]' (number of my children).
Please choose one of the following answers:
Please select only one of the following answers:

- often
- sometimes
- rarely
- never

Has your child taken any medicines to reduce fever in the last 12 months?
Answer this question only if the following conditions are met:
Answer was NOT '0' to question '4 [numberchildren]' (number of my children).
Please choose one of the following answers:
Please select only one of the following answers:

- yes
- no
- do not know

What fever-reducing medicines has your child taken?
Answer this question only if the following conditions are met:
Answer was NOT “0” for question “4 [numberchildren]” (number of my children).
Please choose one of the following answers:
Please select only one of the following answers:

- Paracetamol
- Ibuprophen
- do not know
- Other

Do you reduce fever with naturopathic or homeopathic medicines?
Please select one of the following answers:
Please select only one of the following answers:

- yes ->
- no
- do not know

Please write a comment about your selection

Do you reduce fever by other means (calf compresses, lemon compresses, enemas, etc.)?
Please select one of the following answers:
Please select only one of the following answers:

- yes ->
- no
- do not know

Please write a comment about your selection

Attachment 1 to Gwiasda M, Schwarz S, Büssing A, Jenetzky E, Krafft H, Hamideh Kerdar S, Rathjens L, Boehm K, Martin D. *Chaning knowledge and attitudes about childhood fever: testing a video instruction bevor ist application in a health app.* GMS J Med Educ. 2022;39(2):Doc25. DOI: 10.3205/zma001546
